# Coronary Polyarteritis Nodosa: Clinical Dilemmas in Diagnosis and Management

**DOI:** 10.1016/j.jaccas.2025.103906

**Published:** 2025-07-09

**Authors:** Batool Jamal AbuHalimeh, Stefan Sanger, Laszlo Gobolos, Raghad Abuhalimeh, Lina Abuhalimeh, Houssam Younes

**Affiliations:** a Heart, Vascular and Thoracic Institute, Cleveland Clinic Abu Dhabi, Abu Dhabi, UAE; b University of Jordan, Amman, Jordan

**Keywords:** coronary polyarteritis nodosa, polyarteritis nodosa, vasculitis

## Abstract

**Clinical Conditions:**

A 43-year-old woman presented with multiple coronary vascular wall irregularities, triple-vessel segmental stenosis, coronary aneurysmal dilatation, and coronary collateral arteries. She was diagnosed with polyarteritis nodosa (PAN) with coronary involvement.

**Key Questions:**

What is the approach of management for coronary PAN? What role does a multidisciplinary approach play in optimizing the management and outcomes of patients with coronary PAN?

**Outcomes:**

The patient was treated with aggressive immunotherapy, including high-dose corticosteroids, mycophenolate, and later rituximab, to control inflammation and prevent vascular damage. Antithrombotic therapy (clopidogrel and rivaroxaban) and cardiovascular risk factor management (statins, angiotensin-converting enzyme inhibitors, and smoking cessation) were initiated. Although coronary artery bypass graft was considered because of triple-vessel disease, it was deferred as the patient remained stable. Regular follow-up demonstrated symptomatic improvement.

**Take-Home Messages:**

Coronary PAN requires a multidisciplinary approach for diagnosis and management. Medical therapy often takes precedence, with surgical interventions reserved for selected cases.

## Graphical Abstract

**Figure:**
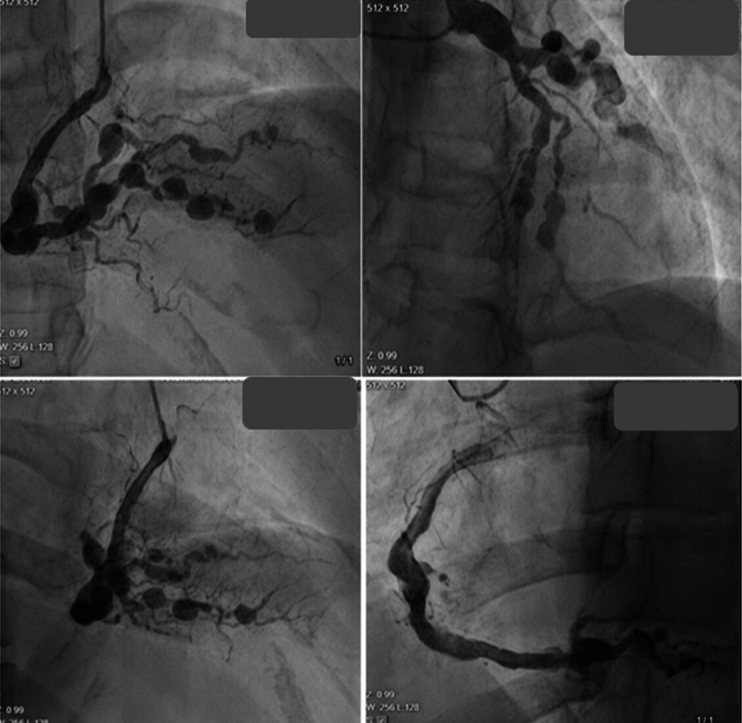

## Case Report

**Figure 1:**
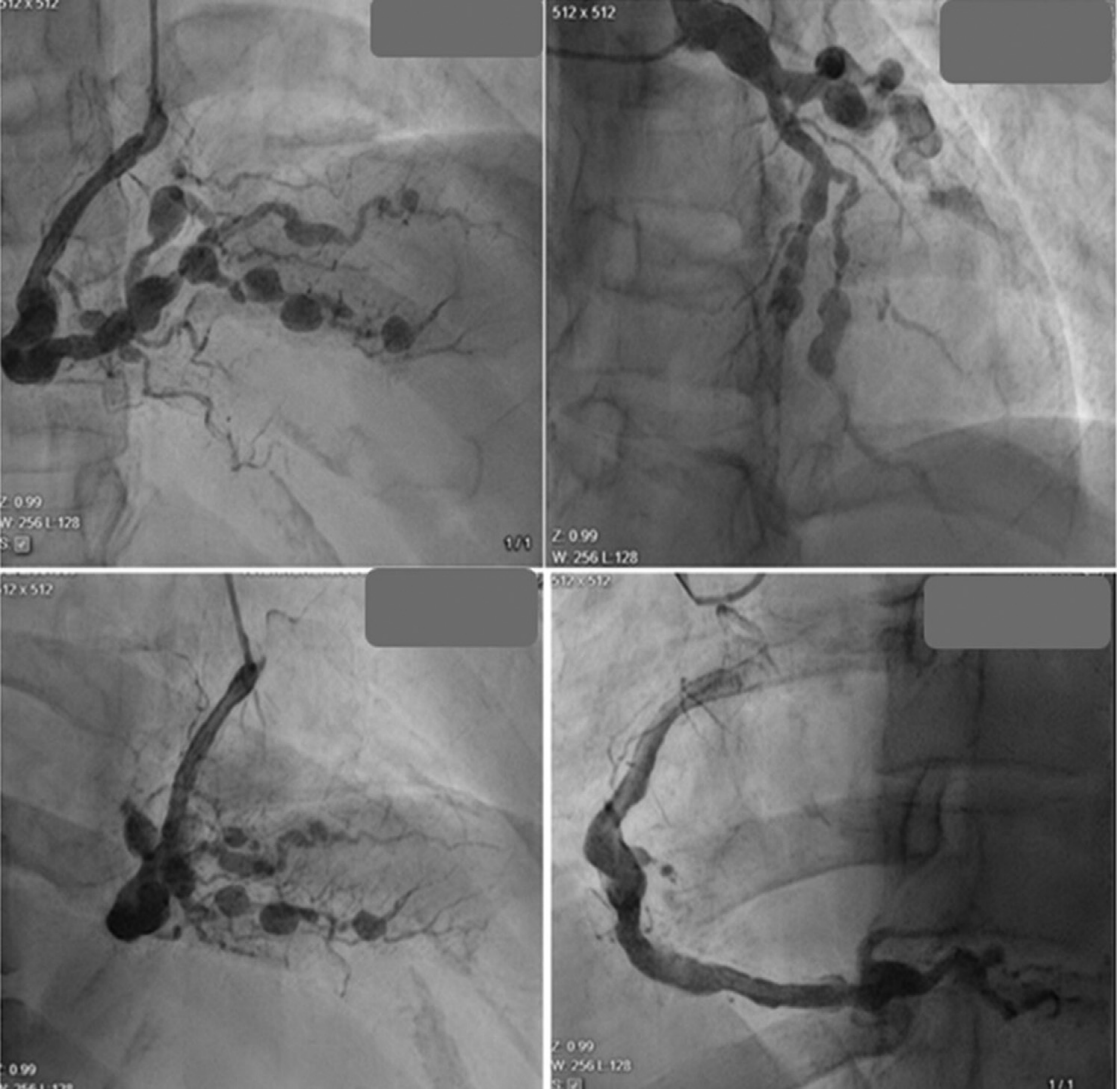
Coronary Angiogram Revealing Irregularities in Coronary Vasculature and Multiple Aneurysm Formation

A 43-year-old woman with a medical history significant for hyperlipidemia presented to the clinic for evaluation of a recurrent report of atypical chest pain. The patient had presented to the emergency department multiple times with this reporting. During her last emergency department visit, she was admitted to the hospital and underwent coronary angiography. The results of angiography revealed multiple coronary vascular wall irregularities, triple-vessel segmental stenosis, and coronary aneurysmal dilatation (Figure 1). In addition, coronary collateral arteries were identified. Eventually, she was referred to cardiac operation for recommendations on surgical management and possible coronary artery bypass graft (CABG).

**Take-Home Messages**

• Coronary polyarteritis nodosa requires a multidisciplinary approach for diagnosis and management.
• Medical therapy often takes precedence, with surgical interventions reserved for selected cases.

On further history taking, the patient stated that she has been complaining of this atypical chest pain for the past 2 years; she described substernal chest pain radiating to her right arm, neck, and jaw. The patient stated that the pain is intermediate and aching in nature, graded as 5/10 in intensity, usually exacerbated by stress or exercise. The patient stated resting as an alleviating factor for the pain. In addition to atypical chest pain, the patient reported a history of intermittent fever, malaise, diffuse arthralgia, myalgia, and difficulty gaining weight. She also reported melena and nonspecific diffuse intermittent abdominal pain. The patient stated that she had been worked up for the above-mentioned symptoms, and the etiology of the chest pain was thought to be secondary to gastroesophageal reflux disease. In addition, she had an endoscopy and colonoscopy in the past few months, which revealed gastritis. The patient denied use of amphetamines, illicit drug use, hepatitis C or B infection, or human immunodeficiency virus exposure/infection.

On physical examination, the patient was found to be underweight with a body mass index of 17.2 kg/m^2^; no skin lesions were noted on examination; neurological examination was insignificant; and carotid, upper, and lower extremity pulses were strong and palpable. Investigations conducted, including laboratory results, revealed normocytic anemia with an elevated reticulocyte count, hematuria, and neutrophilic leukocytosis. Other laboratory results, including serum creatinine levels, muscle enzyme concentrations, liver function studies, erythrocyte sedimentation rate, and C-reactive protein concentrations, were normal. Autoimmune work-up, including antineutrophil cytoplasmic antibodies, antinuclear antibodies, complement components (C3 and C4), cryoglobulins, and serum and urine immunofixation electrophoresis to test for monoclonal gammopathy, was negative. Infectious work-up, including testing for human immunodeficiency virus and hepatitis B virus and hepatitis C virus serologies, was negative.

On the basis of the patient’s symptoms and investigation, large cell vasculitis, infectious disease, and autoimmune conditions (such as systemic lupus erythematosus, rheumatoid vasculitis, systemic sclerosis, drug-induced vasculitis) were excluded. The patient’s coronary angiogram was concerning for polyarteritis nodosa (PAN) vs Kawasaki disease; however, given the patient’s age and lack of involvement of mucocutaneous lymph nodes, PAN was the most likely diagnosis.

The patient was ultimately initiated on an aggressive dose of immunotherapy, including high-dose corticosteroids with mycophenolate, to control inflammation and prevent further vascular damage. In addition, the patient was initiated on clopidogrel and rivaroxaban. Cardiovascular risk factor modifications were addressed and controlled aggressively, including high statin therapy, management of hypertension through angiotensin-converting enzyme inhibitors, and smoking cessation. A few weeks after the diagnosis, rituximab was added to the patient’s management regimen. The patient was evaluated for the possibility of CABG, given triple-vessel disease and coronary aneurysm; however, given the patient was stable, the decision was made to defer surgical management. Regular follow-up of the patient, together with cardiovascular monitoring, was implemented and closely monitored. The patient reported improvement in her symptoms.

## Discussion

We hereby present a case of coronary PAN; this case underscores the formidable challenges encountered in both diagnosing and managing this rare condition. PAN is a rare disease, and when it involves the coronary arteries, its rarity is further compounded.^1^^,^^2^ In addition, coronary PAN may present through nonspecific symptoms that may mimic other clinical conditions including other types of vasculitis, autoimmune disease, infectious disease, myocardial infarctions, and drug-induced coronary vasospasm.^1^ Hence, clinicians may not initially consider PAN in the differential diagnosis of coronary artery disease. Furthermore, the lack of specific diagnostic criteria and reliance on a combination of clinical presentation, laboratory, and imaging findings contribute to the diagnostic dilemma. Traditional diagnostic modalities, such as coronary angiography, may not always reveal the characteristic findings of PAN. Therefore, the rarity, nonspecific presentation of coronary PAN, and lack of diagnostic criteria often lead to delay in diagnosis, which ultimately complicates management and worsens prognosis of this condition. Therefore, a multidisciplinary approach involving expertise of vascular specialists, rheumatologists, cardiologists, cardiac surgeons, and radiologists is essential in such challenging cases to ensure accurate and timely diagnosis.

Management of coronary PAN poses significant therapeutic challenges. There are no evidence-based guidelines to direct appropriate management of coronary PAN; furthermore, data on the management of complications of coronary PAN, such as coronary aneurysms, are based on individual expertise and recommendations.^3^^,^^4^ The systemic nature of PAN necessitates aggressive immunosuppressive therapy to halt disease progression and prevent further organ damage. However, balancing the risks and benefits of immunosuppression, particularly in the context of coronary involvement where the potential for myocardial ischemia and infarction exists, requires careful consideration. Moreover, the optimal duration and intensity of immunosuppressive therapy remain unclear, with limited evidence to guide treatment decisions.

Similarly, the surgical approach to coronary PAN presents a complex area with considerable ambiguity. Whether patients with this condition would benefit from surgical intervention remains uncertain. Although some experts advocate for addressing coronary issues in these patients regardless of their underlying vasculitis, the intricate nature of PAN complicates this decision-making process. For example, in cases involving coronary artery aneurysms, management strategies may include medical therapy, surgical resection, or stent placement.^3^^,^^4^ However, the most appropriate option depends on the patient’s clinical status. In addition, there have been reports of complete resolution of coronary artery aneurysms without intervention. Hence, it is suggested that treatment should be individualized on the basis of various factors such as the patient’s clinical condition, aneurysm size, expansion rate, and associated symptoms.^3^ Aoki et al proposed a tailored approach to treating coronary artery aneurysms, considering factors such as aneurysm size, expansion history, underlying pathophysiology, and symptomatology, to determine the optimal timing and necessity of therapeutic interventions.^4^ However, this approach has not been described in the context of coronary PAN, yet it can still be considered in such a clinical context.

The role of interventional management in PAN with coronary involvement continues to be a subject of debate, as most case reports in literature are controversial.^5^^,^^6^ Therefore, we recommend that each case be treated on the basis of individual risks and management considerations. Management should be tailored to the disease’s extent and specific characteristics. Percutaneous coronary intervention with stenting is generally preferred for occlusive and aneurysmal disease,^6^ whereas CABG is a viable option, particularly for patients with triple-vessel disease.^5^^,^^6^ However, the limited number of reported cases necessitates careful graft selection.^6^ In addition to systemic immunosuppressive therapy, adjunctive measures such as antiplatelet and anticoagulation therapy should be implemented.^5^ Determining the optimal timing for interventions or surgeries remains a considerable challenge in these patients and should be carefully tailored to each individual case.

It is essential to note that standard atherosclerotic risk factors do not accurately predict the risk of coronary disease in these cases.^5^ Importantly, identifying risk factors for coronary involvement, such as new-onset hypertension or celiac involvement, can aid physicians in screening appropriate populations. Furthermore, the management of coronary PAN should include cardiovascular risk modification, including appropriate management of hypertension, diabetes, and hyperlipidemia. In addition, appropriate activity modifications and psychological counseling should be discussed with patients. Nevertheless, close follow-up and monitoring for the development of coronary artery aneurysms, myocardial infarction, and arrhythmias is essential. The long-term prognosis of coronary interventions in PAN depends on disease control, type of intervention, and systemic inflammation. As mentioned earlier, it is preferred for occlusive lesions whereas CABG suits complex cases. Effective immunosuppression, antiplatelet therapy, and regular monitoring are crucial. Limited data highlight the need for individualized, multidisciplinary management.

## Conclusions

The case of coronary PAN sheds light on the intricate diagnostic and therapeutic landscape surrounding this rare condition. The challenges in diagnosing coronary PAN lie in its rarity, diverse clinical presentation, and lack of specific diagnostic criteria. Moreover, managing coronary PAN requires a delicate balance between aggressive immunosuppression to halt disease progression and the potential cardiovascular complications associated with coronary involvement. A multidisciplinary approach involving vascular specialists, rheumatologists, cardiologists, cardiac surgeons, and radiologists is imperative for timely diagnosis and optimal management.

## Funding Support and Author Disclosures

The authors have reported that they have no relationships relevant to the contents of this paper to disclose.
